# Enterotype-Specific Effects of Red Beetroot (*Beta vulgaris* L.) Powder and Betanin on Human Gut Microbiota: A Preliminary Study Based on In Vitro Fecal Fermentation Model

**DOI:** 10.3390/life14111391

**Published:** 2024-10-29

**Authors:** Gwang-Pyo Ko, Hyejun Jo, Jungman Kim, Jeong Seon Kim, Kyung-Hwan Boo, Chang Sook Kim

**Affiliations:** 1 Faculty of Biotechnology, Jeju National University, Jeju 63243, Republic of Korea; rhkdvy1004@gmail.com (G.-P.K.); hyejun758@gmail.com (H.J.); khboo@jejunu.ac.kr (K.-H.B.); 2 Subtropical/Tropical Organism Gene Bank, Jeju National University, Jeju 63243, Republic of Korea; kjm5364@gmail.com; 3 Jeju Institute of Korean Medicine, Jeju 63309, Republic of Korea; 4 Jeju Special Self-Governing Province Agricultural Research & Extension Services, Seogwipo-si 63556, Republic of Korea; kjs0066@korea.kr

**Keywords:** *Beta vulgaris* L., gut microbiota, enterotype, short-chain fatty acid, personalized nutrition

## Abstract

Red beetroots, rich in betanin, may act as prebiotics and impact gut microbiota. Because the human gut microbiota is unique to each person, the effectiveness of prebiotics varies with the enterotype. In this study, we hypothesized that the effects of red beetroot powder (RP) and betanin pigment (BP) would differ depending on the enterotype. Fecal samples from 30 subjects were analyzed and categorized into three enterotypes: *Phocaeicola*, *Prevotella*, and *Bifidobacterium*. Feces were collected from one representative subject from each enterotype cluster for fermentation. Results showed that RP and BP affected microbiota composition and short-chain fatty acid (SCFA) production differently across enterotypes. The *Bifidobacterium* cluster showed significantly reduced alpha diversity, with the direction of change in the gut microbiota composition being different from that of other subjects. Additionally, SCFAs significantly increased, with the highest increase in the *Bifidobacterium* cluster. In this cluster, metabolic pathways related to SCFAs (i.e., starch and sucrose metabolism and glycolysis/gluconeogenesis) were altered. Conversely, *Prevotella*-dominant feces exhibited fewer changes in SCFAs and a lower increase in *Bifidobacterium* abundance than the others. These findings highlight that RP and BP elicit enterotype-specific responses in the gut microbiota composition and SCFA production, emphasizing the importance of enterotypes in personalized nutrition.

## 1. Introduction

Trillions of microorganisms reside in the human intestine, and the intestinal microbiota is often referred to as the second genome [1]. The gut microbiota interacts with the host, contributing significantly to overall health and playing a crucial role in digestion and nutrient absorption. For example, the gut microbiota is involved in metabolic diseases such as obesity, hypertension, diabetes, atopy, and depression [2,3,4,5]. These microbes affect the immune system, break down food to produce nutrients, and metabolize food to produce various metabolites such as essential amino acids, short-chain fatty acids (SCFAs), and vitamins [6]. SCFAs are metabolites produced by the gut microbiota during the fermentation of dietary fiber and non-digestible carbohydrates, boosting the immune system, thereby increasing resistance to infection and inflammation, and affecting the nervous and endocrine systems by acting as signaling molecules [7,8]. Importantly, the composition of the gut microbiota is closely correlated with SCFA production [9]. Specific bacterial groups, such as *Faecalibacterium*, *Bifidobacterium*, and *Bacteroides*, are particularly effective at fermenting dietary fibers, resulting in higher levels of SCFAs. Conversely, dysbiosis—an imbalance in gut microbiota—can lead to decreased SCFA production.

The human gut microbiota is strongly influenced by a variety of factors, including age, nutritional status, and geographic environment. Notably, enterotypes are defined as various clusters of the human gut microbiota, categorized based on core bacteria such as *Bacteroides*, *Prevotella*, *Ruminococcus*, *Bifidobacterium*, and *Faecalibacterium* [10,11,12]. Interestingly, recent research suggests that individuals exhibit different metabolic responses, even when consuming the same diet, depending on their enterotypes [13]. Accordingly, enterotypes are important indicators of personalized nutrition. Personalized nutrition is a nutritional strategy that considers each individual’s genetics, health status, lifestyle, and dietary habits to customize their nutrient requirements and intake recommendations. Recently, microbiome analysis methods, such as enterotyping, have been introduced to minimize individual differences and identify the effects of diet [14]. Thus, predicting dietary responses based on enterotypes can help develop personalized nutritional strategies that consider an individual’s microbiome structure, which is expected to play an important role in personalized medicine and nutrition.

Red beetroot (*Beta vulgaris* L.) is a vegetable rich in phytochemicals such as dietary fiber, polyphenols, and betalains, making it popular worldwide for its health benefits. The main betalain in red beetroot is betanin, which belongs to the betacyanin subgroup and is the only betacyanin approved as a food additive by the European Union and the Food and Drug Administration (FDA) [15,16]. Betanin possesses anti-inflammatory, antioxidant, and immune-regulating effects [17]. Remarkably, these beneficial effects may be mediated by gut microbiota [18]. Enzymes produced by gut microbiota, such as β-glucosidase and glycoside hydrolases, can metabolize phytochemicals, including dietary fiber, polyphenols, and betanin, into SCFAs [19,20,21]. A previous study reported that the consumption of red beetroot juice increases SCFAs, potentially associated with an increase in betacyanin catabolites by gut microbiota [22]. Additionally, consuming whole beetroot has been shown to lower systolic blood pressure and enhance SCFA production through the regulation of intestinal microbiota in elderly individuals [23]. Thus, red beetroot and betanin can be utilized as prebiotics. However, it is important to note that representative bacteria, such as *Bifidobacterium* and *Bacteroides*, which produce microbial enzymes capable of metabolizing these substances in the gut, are core bacteria of specific enterotypes [24,25]. Therefore, the response to red beetroot and betanin may vary depending on the enterotype.

This study aimed to provide basic information for the development of personalized prebiotic materials by investigating the effects of red beet powder (RP) and betanin pigment (BP) on the gut microbiota composition and short-chain fatty acid production based on enterotype using a gastrointestinal digestion and fecal fermentation (GID-FF) model. Our findings highlight that RP and BP elicit enterotype-specific responses in the gut microbiota composition and SCFA production, emphasizing the importance of enterotypes in personalized nutrition.

## 2. Materials and Methods

### 2.1. Reagents and Materials

RP was prepared using beetroot grown in the Jeju Special Self-Governing Province Agricultural Research and Extension Service. BP, α-amylase, pepsin, pancreatin, and standard SCFAs (acetate, propionate, and butyrate) were purchased from Sigma-Aldrich (St. Louis, MO, USA).

### 2.2. Test Subjects and Fecal Sample Collection

This study was approved by the Institutional Review Board of Jeju National University (approval number: JJNU-IRB-2024-026) and registered with the Clinical Research Information Service (CRIS: https://cris.nih.go.kr/cris/index/index.do, Clinical Trial Registry Number: KCT0009657, accessed on 26 July 2024). We recruited 30 healthy subjects (17 men and 13 women) for enterotype analysis (primary outcome) with no underlying medical conditions such as diabetes, cancer, liver disease, or history of gastrointestinal disease. Based on this, we selected one subject per enterotype to assess the impact on gut microbiota (secondary outcomes). None of the individuals had been treated with antibiotics for at least 3 months before sample collection. Fecal samples were stored at −20 °C immediately upon collection and subsequently transported to the lab for storage at −80 °C. Fecal samples were collected immediately for fermentation.

### 2.3. In Vitro Gastrointestinal Digestion (GID) and Fecal Fermentation (FF)

GID-FF was performed as previously described, with several modifications [26,27]. In the salivary digestion phase, 0.5 g of RP and BP were mixed with 4.5 mL of PBS (pH 7), 3.5 mL of simulated salivary fluid (SSF), 0.5 mL of alpha-amylase (1500 U/mL make-up SSF), 25 μL of 0.3 M CaCl_2_, and 975 μL of distilled water (DW). This was incubated at 37 °C for 2 min at 150 rpm. In the gastric digestion phase, the salivary digestion product was mixed with 7.5 mL of simulated gastric fluid (SGF), 1.6 mL of pepsin (25,000 U/mL in SGF), 5 μL of 0.3 M CaCl_2_, and 695 μL of DW, adjusted to pH 2 using 4 N HCl, and incubated at 37 °C for 2 h at 100 rpm. In the intestinal digestion phase, the gastric digestion product was mixed with 11 mL of simulated intestinal fluid (SIF), 5 mL of pancreatin (800 U/mL in SIF), 2.5 mL of bile salt, 40 μL of CaCl_2_, and 1.31 mL of DW, adjusted to pH 7 using 1 M NaOH and incubated at 37 °C for 2 h at 100 rpm.

The day before the fecal fermentation experiment, PBS and the basal culture medium were placed overnight in an anaerobic chamber (90% N_2_, 5% H_2_, and 5% CO_2_) to remove oxygen. The fecal samples were collected in a sterilized 50-mL conical tube and immediately transferred into an anaerobic chamber upon receipt. The transferred fecal sample was homogenized with PBS (20% *w*/*v*) using a vortex and sieved through pore sizes of 250, 150, and 25 μm to filter the residue. For fecal fermentation, 1.2 mL of basal culturing medium and 150 μL of GID product (10%) were dispensed into each well of a 96-well deep plate and inoculated with 150 μL of sieved feces in PBS (10%). The fermentation was performed in triplicates at 100 rpm for 6 h at 37 °C in an anaerobic chamber using digital shakers.

### 2.4. Analysis of Microbial Community

An amount of 1.5 mL of the sample was centrifuged at 13,000 rpm for 5 min at 4 °C. The pellet was used for genomic DNA extraction using a QIAamp PowerFecal Pro DNA Kit (QIAamp, Germantown, MD, USA). To analyze the microbial community in the samples, polymerase chain reaction (PCR) was used to amplify the V3–V4 hypervariable region of the 16S rRNA gene. The PCR product was purified, and individual indices were added to the amplicons for each sample using PCR once again. After purifying the PCR products in the same manner, sequencing was performed using the Illumina MiSeq platform (Illumina, San Diego, CA, USA). All sequencing procedures were performed by Macrogen, Inc. (Seoul, Republic of Korea).

Sequencing data were analyzed according to the MOTHUR SOP guidelines using the MOTHUR software version 1.47.0 (https://mothur.org/wiki/miseq_sop/, accessed on 26 July 2024) [28]. Briefly, raw reads obtained from Miseq were assembled using “make.contigs” and aligned to the SILVA database version 138 using “align.seqs” [29]. Rare sequences and singletons were removed using “pre.cluster” and “spit.abun,” potential chimeric sequences were read using “chimera vserach” [30], taxonomy classification of bacteria was read with Ribosome database project version 18 using “classify.seqs” [31], and undesired taxa sequences (i.e., chloroplast, mitochondria, unknown, and Eukaryota) were eliminated using “remove.lineage” and clustered based on OptiClust algorithms with 97% similarity using “opti.clust” [32]. The number of reads was normalized to 20,000 for downstream analysis. Enterotype classification was performed using “get.communitytype” using the k-means clustering algorithm with tree analysis.

The α-diversity indices for richness and evenness were calculated based on Chao and Shannon within MOTHUR. The β-diversity indices for non-metric multidimensional scaling (NMDS) analysis evaluated the differences in each group. The metabolic pathways of fecal microbiota were estimated using the Phylogenetic Investigation of Communities by Reconstruction of Unobserved States (PICRUSt2) version 2.4.2 [33], and KO abundances were converted to KEGG pathway abundances using the ggpicrust2 package version 1.7.3 in R [34].

### 2.5. SCFAs Extraction and Quantification

An amount of 1.5 mL of the sample was centrifuged at 13,000 rpm for 5 min at 4 °C, and the supernatant was used for SCFA extraction. An amount of 200 μL of the supernatant was added to 800 μL of absolute methanol and homogenized for 2 min. The pH of the mixture was adjusted to 2–3 using HCl, and the mixture was incubated at room temperature for 10 min, with frequent homogenization every 3 min. Finally, the mixture was filtered through a membrane with a pore size of 0.45 μm.

Quantification of SCFAs (acetate, propionate, and butyrate) was conducted using Gas Chromatography (GC2010, Shimadzu, Japan) with a flame ionization detector (FID) on Split mode (10:1 ratio) using a DB-FFAP column (30 m × 0.25 μm × 0.25 μm, Agilent, Santa Clara, CA, USA). The temperature of the inlet and FID was maintained at 230 °C and 280 °C, respectively, and 1 μL of the filtered mixture was injected into the column. During operation, the column oven had the following temperature: 80 °C for 3 min, and then gradually increased to 200 °C at a rate of 15 °C/min. At 200 °C, it was held for another 3 min, then increased to 230 °C at a rate of 5 °C/min, and finally held for 10 min.

### 2.6. Statistical Analysis

Data is expressed along with standard deviation. SCFAs and α-diversity indices were evaluated using analysis of variance (ANOVA). Analyses of molecular variance (AMOVA), NMDS, and enterotype clustering were performed using the Bray–Curtis coefficient [35]. The differential abundance of microorganisms between groups was calculated using a linear discriminant analysis effect size (LEfSe) based on the Kruskal–Wallis (KW) sum-rank test [36]. Additionally, the significant differential abundance of the predicted microbial metabolic activities between groups was investigated using a two-sided Welch’s *t*-test via STAMP version 2.1.3 [37]. Correlation analysis between the bacterial genera and SCFAs was performed using Pearson’s rank correlation coefficient.

## 3. Results

### 3.1. Enterotype Analysis

**Figure 1:**
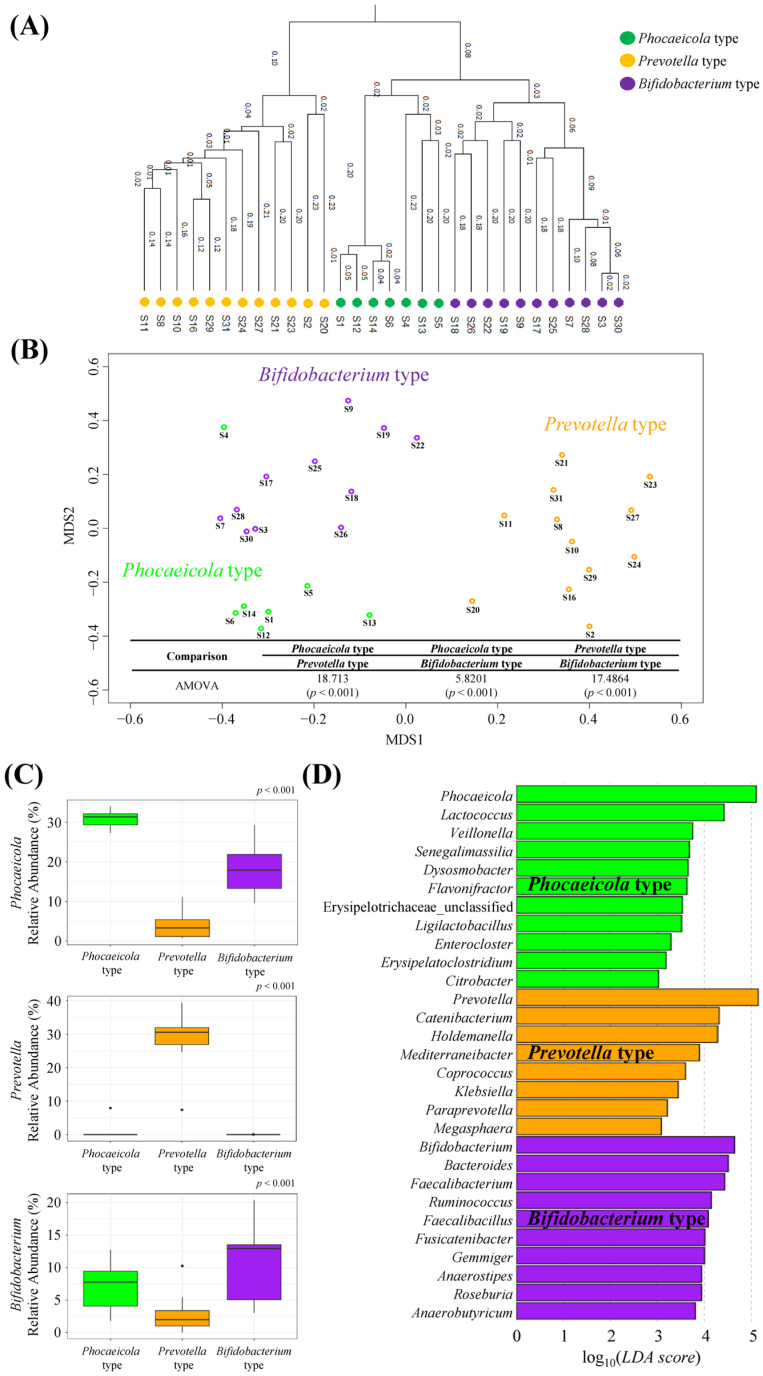
Fecal microbial enterotype clustering. (**A**) Tree analysis, (**B**) non-metric multidimensional scaling (NMDS) with analysis of molecular variance (AMOVA), (**C**) relative abundance of *Phocaeicola*, *Prevotella*, and *Bifidobacterium* in each enterotype, and (**D**) linear discriminant analysis (LDA) effect size (LEfSe) of three different enterotypes at the genus level. (*p* < 0.05, LDA score > 3).

Enterotype analysis was performed on fecal samples collected from 30 participants (participants 1–30). The gut microbiota of the 30 subjects was categorized into three types. Each enterotype not only exhibited a relatively scattered distribution in the Tree and NMDS plots but also demonstrated statistically significant differences, primarily driven by the genera *Phocaeicola*, *Prevotella*, and *Bifidobacterium* as the core microbiota (*p* < 0.05, Figure 1A–C). We designated enterotypes according to the core microbiota as the *Phocaeicola* (*n* = 7), *Prevotella* (*n* = 12), and *Bifidobacterium* (*n* = 11) types. Analysis of 30 fecal samples at the phylum level revealed that the *Phocaeicola* type had a higher relative abundance of Proteobacteria than other types. Compared to the other types, the *Prevotella* type showed a higher relative abundance of Bacteroidetes, whereas the *Bifidobacterium* type had a higher relative abundance of Firmicutes and Actinobacteria (Figure S1). Furthermore, we examined the microbiota with significantly different genera by enterotype, in addition to the core microbiota, which is summarized in Figure 1D. The *Phocaeicola* type exhibited enrichment of 11 genera, such as *Lactococcus* and *Veillonella*, whereas the *Prevotella* type demonstrated enrichment of 8 genera, including *Catenibacterium* and *Holdemanella*. In contrast, the *Bifidobacterium* cluster was enriched with 10 genera, including *Bacteroides*, *Ruminococcus*, and *Faecalibacterium*. Characteristics such as age and BMI of the subjects in each type were not significantly different (*p* > 0.05, Table S1).

### 3.2. Microbial Communities of GID-FF-Treated Fecal Samples

**Figure 2:**
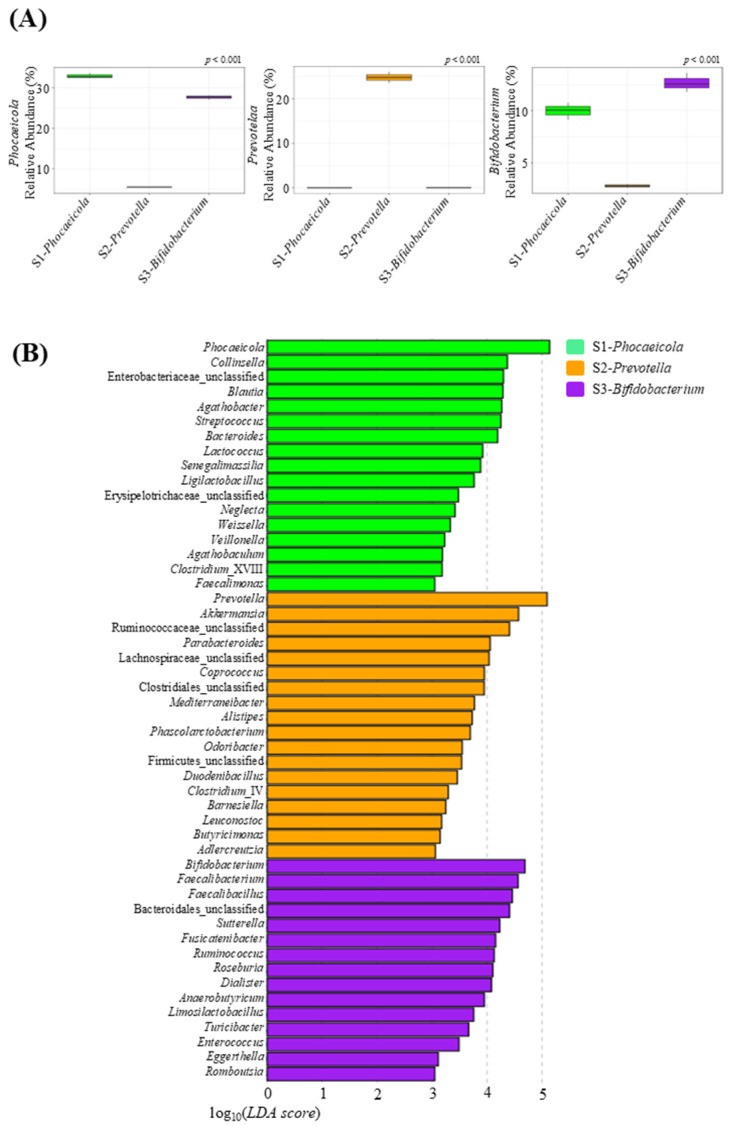
The fecal microbiota of the subjects selected based on enterotype before fecal fermentation. (**A**) relative abundance of *Phocaeicola*, *Prevotella*, and *Bifidobacterium* in each subject, and (**B**) linear discriminant analysis (LDA) effect size (LEfSe) at the genus level (*p* < 0.05, LDA score > 3).

For GID-FF, fecal samples were collected from one subject of each type from the 30 subjects (Subject 1, Subject 2, and Subject 3 were selected), and the preferentially selected samples (labeled S1-*Phocaeicola*, S2-*Prevotella*, and S3-*Bifidobacterium*) were checked to ensure that they were representative of each type. As shown in Figure S2, the S1-*Phocaeicola* sample belonged to the *Phocaeicola* type, whereas S2-*Prevotella* and S3-*Bifidobacterium* belonged to the *Prevotella* and *Bifidobacterium* types, respectively. In addition, the abundance of *Phocaeicola*, *Prevotella*, and *Bifidobacterium*, which are the core bacteria in each selected subject, was consistent (Figure 2A). Therefore, the selected sample remained representative of each enterotype.

At the genus level, 17, 18, and 15 genera were enriched in S1-*Phocaeicola*, S2-*Prevotella*, and S3-*Bifidobacterium* samples, respectively (Figure 2B). In particular, *Lactococcus*, *Senegalimassilia*, *Ligilactobacillus*, and *Veillonella*, which are abundant in the *Phocaeicloa* type, were enriched in the S1-*Phocaeicloa* sample, whereas *Coprococcus* and *Mediterraneibacter*, which are abundant in the *Prevotella* type, were enriched in the S2-*Prevotella* sample. Similarly, *Faecalibacterium*, *Faecalibacillus*, *Fusicatenibacter*, *Ruminococcus*, *Roseburia*, and *Anaerobutyricum*, which are abundant in the *Bifidobacterium* enterotype, were enriched in the S3-*Bifidobacterium* sample. At the phylum level, Proteobacteria, which had a higher relative abundance in the *Phocaeicloa* type, and Actinobacteria, which had a higher relative abundance in the *Bifidobacterium* type, were both enriched in S1-*Phocaeicloa*, and Verrucomicrobia were enriched in S2-*Prevotella* (Figure S3).

### 3.3. Enterotype-Specific Effects of RP and BP on Gut Microbiota

**Figure 3:**
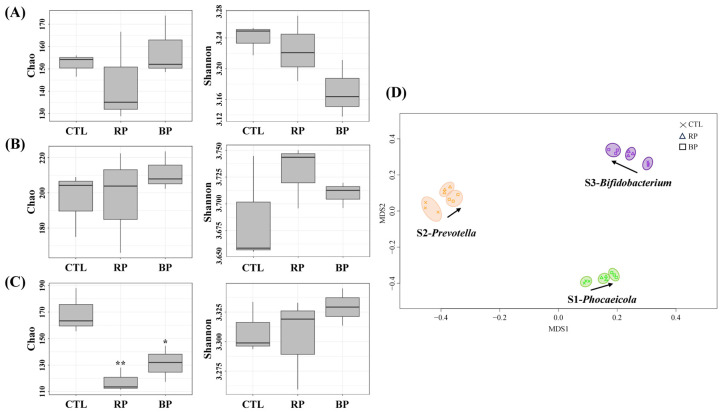
Analysis of α and β diversity by enterotype following RP and BP fermentation. (**A**) S1-*Phocaeicloa*, (**B**) S2-*Prevotella*, (**C**) S3-*Bifidobacterium*, and (**D**) non-metric multidimensional scaling (NMDS). CTL, negative control (without fermentable substrate); RP, red beet powder; BP, betanin pigment. * *p* < 0.05, ** *p* < 0.01.

To investigate the effects of RP and BP on the gut microbiota based on enterotype, GID-FF was performed on S1-*Phocaeicola*, S2-*Prevotella*, and S3-*Bifidobacterium* fecal samples treated with RP and BP. The alpha and beta diversities of the fecal samples after fermentation are shown in Figure 3. We compared the Chao and Shannon indices, which represent species richness and evenness of the gut microbiota, respectively. When the S1-*Phocaeicola* type sample was treated with RP and BP, the Chao indices were 143.52 ± 11.69 and 158.19 ± 7.90 and the Shannon indices were 3.18 ± 0.02 and 3.16 ± 0.03, respectively, while for the S2-*Prevotella* type sample, the Chao indices were 197.44 ± 16.61 and 211.28 ± 6.36 and the Shannon indices were 3.74 ± 0.05 and 3.71 ± 0.01, respectively. For the S3-*Bifidobacterium* sample, the Chao indices were 117.76 ± 5.21 and 131.33 ± 7.83, and the Shannon indices were 3.30 ± 0.01 and 3.32 ± 0.02, respectively. Overall, the Chao index was significantly reduced by the RP and BP only in the S3-*Bifidobacterium* sample (*p* < 0.05), whereas the Shannon index showed no significant changes in any of the samples (*p* > 0.05). In the NMDS, no significant changes in the gut microbiota were observed after fecal fermentation with RP and BP (*p* > 0.05); however, the distribution tended to be scattered by RP and BP treatment (Figure 3D). In addition, the S3-*Bifidobacterium* cluster exhibited a different direction in gut microbiota changes compared to the other samples.

**Figure 4:**
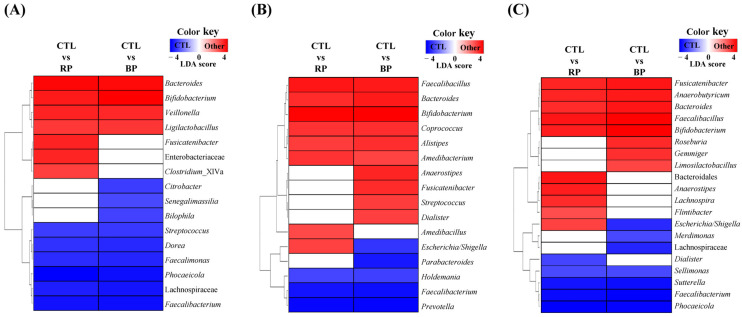
Significantly different relative abundance at the genus level by enterotype following RP and BP fermentation. (**A**) S1-*Phocaeicloa*, (**B**) S2-*Prevotella*, and (**C**) S3-*Bifidobacterium*. Significantly different relative abundance was examined using the liner discriminant analysis (LDA) effect size (LEfSe) at the genus level (*p* < 0.05, LDA score > 3). CTL, negative control (without fermentable substrate); RP, red beet powder; BP, betanin pigment.

Changes in the microbial composition were assessed to determine the effects of RP and BP on the gut microbiota of each sample. The major phyla Firmicutes and Bacteroidetes did not show significant changes, whereas Actinobacteria and Proteobacteria exhibited significant alterations (*p* < 0.05; Table S2). At the genus level, the RP and BP treatments significantly changed the abundance of gut microbes (Figure 4). In S1-*Phocaeicola*, a total of 16 bacteria were significantly altered by RP and BP, with *Bacteroides*, *Bifidobacterium*, *Veillonella*, and *Ligilactobacillus* increasing in both, while *Streptococcus*, *Dorea*, *Faecalimonas*, *Phocaeicola*, Lachnospiraceae, and *Faecalibacterium* decreased (*p* < 0.05). Similarly, in S2-*Prevotella*, a total of 16 bacteria were significantly altered by RP and BP, with *Faecalibacillus*, *Bacteroides*, *Bifidobacterium*, *Coprococcus*, *Alistipes*, and *Amedibacterium* increasing in both, whereas *Holdemania*, *Faecalibacterium*, and *Prevotella* decreased (*p* < 0.05). In the case of S3-*Bfidobacterium*, a total of 20 bacteria were significantly changed by RP and BP, with *Fusicatenibacter*, *Anaerobutyricum*, *Bacteroides*, *Faecalibacillus*, and *Bifidobacterium* increasing in both, whereas *Sellimonas*, *Sutterella*, *Faecalibacterium*, and *Phocaeicola* decreased (*p* < 0.05). Among the bacteria that were increased by RP and BP, *Bacteroides* and *Bifidobacterium* increased in all subjects, whereas *Faecalibacterium* decreased. However, the rate of increase in *Bifidobacterium* was the lowest in the S2-*Prevotella* sample and the highest in the S3-*Bifidobacterium* sample (Table S3). In contrast, *Phocaeicola*, which was the core microbiota of the enterotype, was significantly reduced in the S1-*Phocaeicola* and S3-*Bifidobacterium* samples, whereas *Prevotella* was only present in the S2-*Prevotella* sample and was reduced.

### 3.4. Enterotype-Specific Effects of RP and BP on SCFA Production

**Figure 5:**
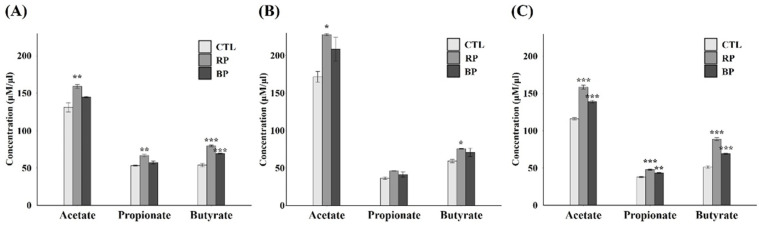
Amount of SCFAs by enterotype following RP and BP fermentation. (**A**) S1-*Phocaeicloa*, (**B**) S2-*Prevotella*, and (**C**) S3-*Bifidobacterium*. CTL, negative control (without fermentable substrate); RP, red beet powder; BP, betanin pigment; SCFA, short-chain fatty acid. * *p* < 0.05, ** *p* < 0.01, *** *p* < 0.001.

Figure 5 shows the difference in SCFA production between the subjects after fermentation. Acetate and propionate in the S1-*Phocaeicola* samples significantly increased in RP (*p* < 0.05), whereas butyrate increased in both RP and BP (*p* < 0.05). In the S2-*Prevotella* sample, fermentation with RP significantly increased the acetate and butyrate levels (*p* < 0.05), whereas no change was observed after fermentation with BP (*p* > 0.05). In contrast, the S3-*Bifidobacterium* sample showed significant increases in acetate, propionate, and butyrate levels in both RP and BP groups (*p* < 0.05).

**Figure 6:**
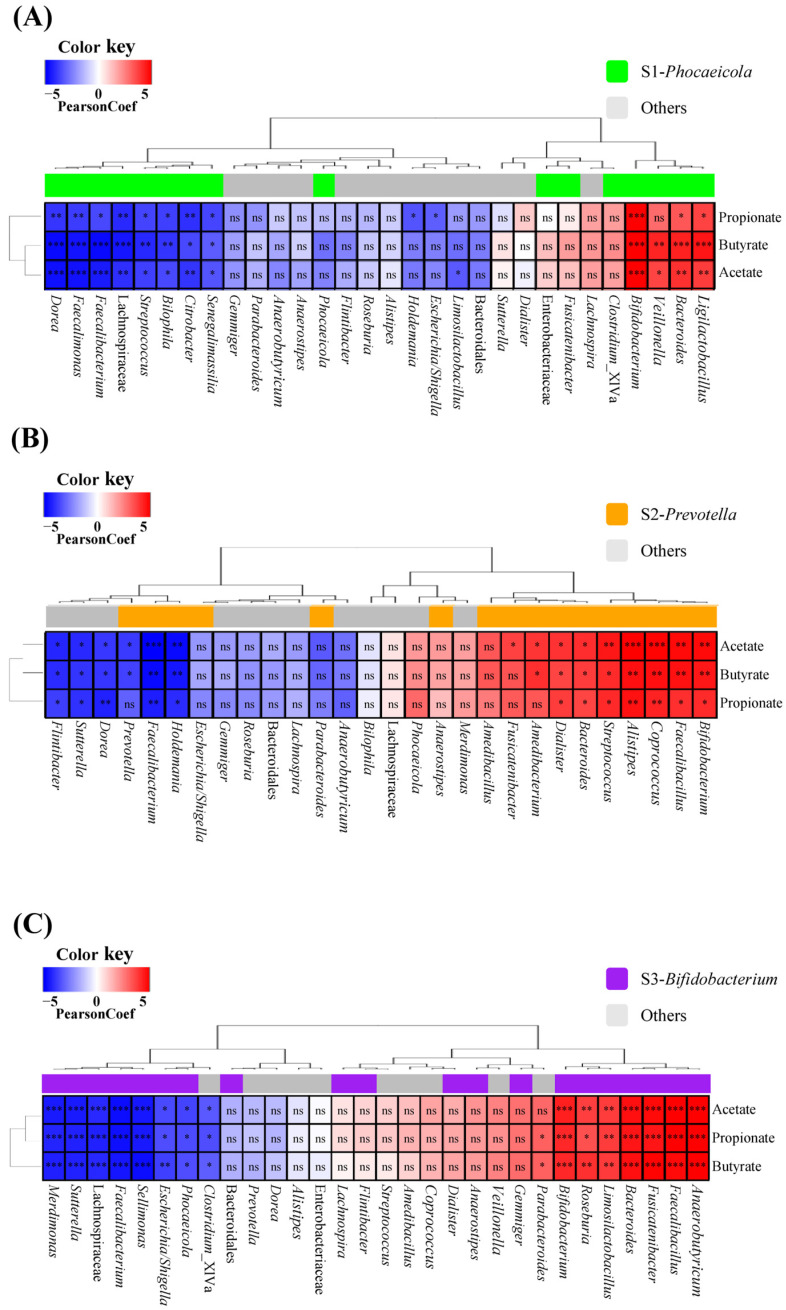
Pearson correlation analysis between fecal microbiota and SCFAs by enterotype. (**A**) S1-*Phocaeicloa*, (**B**) S2-*Prevotella*, and (**C**) S3-*Bifidobacterium*. ns, non-significant; SCFA, short-chain fatty acid. * *p* < 0.05, ** *p* < 0.01, *** *p* < 0.001.

Furthermore, to investigate the association between SCFA production in each subject and their gut microbiota, Pearson correlation analysis was performed between the microbiota and SCFAs that were significantly altered by RP and BP (Figure 6). In general, across all samples, all SCFAs were positively correlated with *Bacteroides* and *Bifidobacterium*, whereas they were negatively correlated with *Facecalibacterium*. Meanwhile, in the S1-*Phocaeicola* sample, *Veillonella* and *Ligilactobacillus*, which were increased by RP and BP, were positively correlated with most SCFAs, whereas *Streptococcus*, *Dorea*, *Faecalimonas*, and Lachnospiraceae, which were decreased by RP and BP, were negatively correlated with all SCFAs. The genera *Faecalibacillus*, *Coprococcus*, *Alistipes*, and *Amedibacterium*, which were increased by RP and BP in the S2-*Prevotella* sample, were positively correlated with most SCFAs, while the genera *Holdemania* and *Prevotella*, which were decreased by RP and BP, were negatively correlated with SCFAs. For the S3-*Bifidobacterium* sample, the genera *Fusicatenibacter*, *Anaerobutyricum*, and *Faecalibacillus*, which increased in both the RP and BP, were positively correlated with all SCFAs, whereas *Sellimonas*, *Sutterella*, and *Phocaeicola*, which decreased in both the RP and BP, were negatively correlated. Importantly, certain microorganisms of each enterotype were positively correlated with SCFA production. For example, *Ligilactobacillus* and *Veillonella* were only positively correlated with SCFA in the S1-*Phocaeicola* sample, while *Coprococcus* and *Alistipes* were only positively correlated in the S2-*Prevotella* sample. Furthermore, the genera *Anaerobutyrticum* and *Roseburia* positively correlated with all SCFAs in the S3-*Bifidobacterium* sample.

### 3.5. Enterotype-Specific Effects of RP and BP on Microbial Metabolic Activities

**Figure 7:**
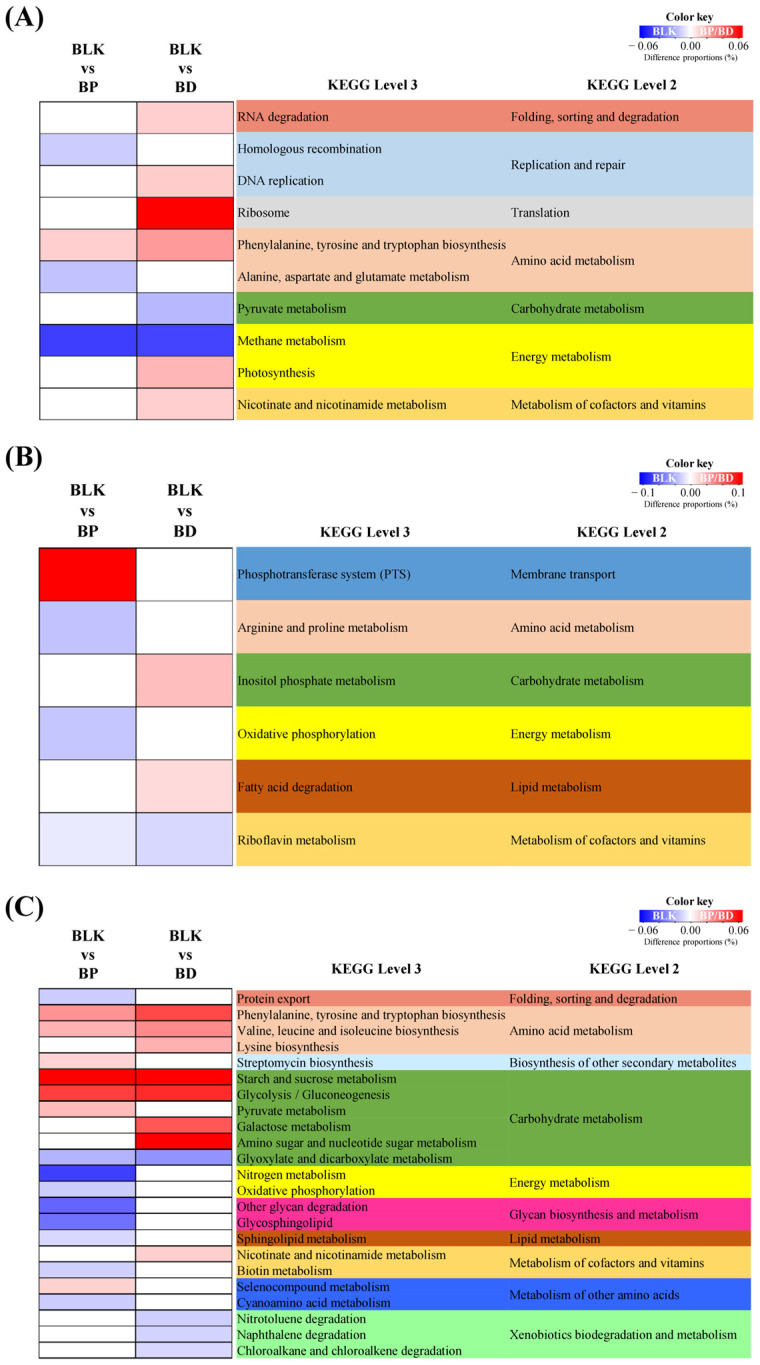
Significantly different relative abundance of PICRUSt2-Predicted microbial metabolic activities by enterotype at Level 3 of KEGG pathway following BP and BD fermentation. (**A**) S1-*Phocaeicloa*, (**B**) S2-*Prevotella*, and (**C**) S3-*Bifidobacterium*. Significantly different relative abundance was calculated using Welch’s *t*-test (*p* < 0.05, Difference proportions > 0.01). CTL, negative control (without fermentable substrate); RP, red beet powder; BP, betanin pigment.

Microbial metabolic activities were predicted using PICRUSt2 by comparing the microbiota function against sequencing data and the KEGG database. Figure S4 shows the pathways significantly altered by RP and BP at KEGG Level 2 for each participant. In the S1-*Phocaeicola* sample, no significant alterations of the pathways were observed, whereas in the S2-*Prevotella* sample, the metabolism of cofactors and vitamins was consistently reduced. In contrast, in the S3-*Bifidobacterium* sample, the metabolism of carbohydrates, terpenoids, and polyketides was increased by RP and BP, whereas energy metabolism, cell growth, and cell death were decreased. In the S2-*Prevotella* sample, the decrease in cofactor and vitamin metabolism was primarily due to riboflavin metabolism. In the S3-*Bifidobacterium* sample, the increase in carbohydrate metabolism was mainly due to glycolysis/gluconeogenesis and the metabolism of starch, sucrose, pyruvate, galactose, amino sugars, nucleotide sugars, glyoxylates, and dicarboxylates (Figure 7B,C). In contrast with the results at KEGG level 2, at level 3 in the S1-*Phocaeicaola* sample, methane metabolism and phenylalanine, tyrosine, and tryptophan biosynthesis were significantly changed by RP and BP (Figure 7A).

## 4. Discussion

In the current study, we aimed to investigate the in vitro enterotype-specific changes in the gut microbiota and SCFAs using RP and BP. We confirmed the hypothesis that the effects of RP and BP on the gut microbiota are enterotype-specific. Specifically, it depended on the relative abundance of *Bifidobacterium* and *Prevotella*. The results of the present study emphasize the importance of enterotypes in personalized nutrition.

We first analyzed the gut microbiota of 30 subjects and then selected one subject per enterotype for fecal fermentation. Three enterotypes were identified, and it was confirmed that the subjects selected for each enterotype truly represented their respective enterotypes. The enterotype is commonly divided into *Prevotella*, *Bacteroides*, and *Ruminococcus* types [10]; however, our results showed that it was divided into *Phocaeicola*, *Prevotella*, and *Bifidobacterium* types in our subjects. Lu et al. reported that enterotypes can vary depending on diet, country, regional environment, and other factors [38]. Previous studies have shown that the gut microbiota of Africans is distributed between *Prevotella* and *Bacteroides*, whereas *Bifidobacterium* and *Bacteroides* are the core microbiota in Asian and Western populations, respectively [12]. This indicates that the enterotype may vary depending on the sample ranges and that people in each region may have an enterotype distribution with corresponding regional characteristics. Therefore, as shown in Figure 1, *Phocaeicola*, *Prevotella*, and *Bifidobacterium* could be representative enterotypes of the core bacteria.

The diverse gut microbiota interact with each other to perform various physiological, metabolic, and immunological functions. Alpha diversity is used to describe the microbial diversity within an ecosystem. In this study, Shannon (evenness, referring to the number or proportion of bacteria within an ecosystem) and Chao (richness, referring to the total number of microbial taxa within an ecosystem) indices were used to evaluate the effects of RP and BP on alpha diversity. After 6 h of fermentation, the Chao index decreased only in S3-*Bifidobacterium* samples treated with RP and BP. A previous study reported that easily fermentable substrates promote the growth of certain gut microbiota, significantly reducing alpha diversity [39,40]. Furthermore, the NMDS analysis showed that the S3-*Bifidobacterium* sample had different directions of RP- and BP-induced changes in the gut microbiota compared to other subjects, similar to studies showing that different enterotypes or subjects have different gut microbiota altered by additives [41,42]. Thus, RP and BP induced changes in the gut microbiota depending on the enterotype, indicating that they can affect host health in a short time, especially in the S3-*Bifidobacterium* sample.

RP and BP affected the abundance of different bacteria in each participant at the genus level, including increases in potentially beneficial bacteria and decreases in potentially harmful bacteria. *Ligilactobacillus* (formerly known as *Lactobacillus*) is a classic probiotic [43], and *Veillonella* helps maintain a healthy gut environment by producing acetate and propionate from lactic acid produced by *Lactobacillus* species [44]. Conversely, *Dorea* was positively associated with body weight, waist circumference, and BMI in overweight/obese subjects [45]. The abundances of these three microbes were altered in the S1-*Phocaeicola* sample. Meanwhile, in the S2-*Preovtella* sample, *Coprococcus* and *Alistipes* increased with RP and BP, whereas *Anaerostipes* increased only with BP. It has been reported that *Coprococcus* alleviates colitis by regulating the gut microbiota and immunoglobulin A [46]. *Alistipes* have been shown to negatively correlate with several inflammatory factors such as LPS and TNF-α [47]. Also, these two bacteria can produce SCFAs [48,49]. In contrast, in the S3-*Bifidobacterium* sample, *Anaerobutyricum*, *Limosilactobacillus*, and *Roseburia* increased, whereas *Sutterella* decreased. *Sutterella* is a pathogen that worsens the immune system and increases susceptibility to intestinal diseases [50], whereas *Anaerobutyricum* (known as *Eubacterium*) is a candidate next-generation probiotic that provides energy to the host and interacts with the immune system by producing SCFAs [51,52]. *Limosilactobacillus* and *Roseburia* regulate barrier homeostasis and cytokine release by producing SCFAs [53,54]. Overall, RP and BP contributed to an increase in beneficial bacteria and a decrease in pathogenic bacteria; however, the effects were different for each enterotype. In particular, the bacteria that increased in each subject were the dominant bacteria in individuals, and these results confirmed the intestinal-specific response to RP and BP from the perspective of the gut microbiota. Additionally, *Bifidobacterium*, *Bacteroides*, *Fusicatenibacter* and *Anaerostipes*, which were elevated in more than two subjects, produce SCFAs and affect host health [55,56,57,58].

The same dietary substrate has been shown to result in different levels of SCFA changes among the enterotypes. It has been shown that fecal fermentation of fibers, such as fructooligosaccharides, sorghum bran, and corn arabinoxylan, significantly increases the total SCFAs and propionate in the *Prevotella* type compared to the *Bacteroides* type [39]. In addition, a previous study using marine oligosaccharides and polysaccharides, including alginate and carrageenan, revealed that total SCFAs and butyrate levels were higher in the *Bacteroides* type than in other enterotypes [59,60]. While most studies on responses to dietary substrates have focused on in vitro experiments, in vivo experiments have also shown that dietary intervention with capsaicin caused a significant increase in butyrate levels in the *Bacteroides* type after intake by the subjects [61]. In this study, there was a tendency to increase SCFAs in all subjects, as well as an increase in SCFA producers and associated bacteria; however, there were differences in the SCFA changes in each subject. The S2-*Prevotella* sample showed fewer changes in SCFAs compared to the other samples, with no significant change caused by BP, whereas the S3-*Bifidobacterium* sample showed a significant increase in all SCFAs owing to the RP and BP treatment. *Prevotella* produces SCFAs through carbohydrate metabolism in the gut [39]. Previous studies have suggested that *Prevotella* is negatively correlated with *Bifidobacterium* and that *Bifidobacterium* growth may be affected by the enterotype [62]. A clinical study in which subjects were administered red beet juice showed increased *Bifidobacterium* abundance and decreased *Prevotella* abundance [22]. Furthermore, *Bifidobacterium* possesses active ingredients involved in enzymatic deglycosylation, which involves the transformation of betanin [21], and betacyanin complexes in the gut of pigs have been shown to increase *Bifidobacterium* [25]. Here, *Bifidobacterium* increased in all subjects after RP and BP fecal fermentation, but the relative abundance and ratio of increase were lowest in the S2-*Prevotella* sample and highest in the S3-*Bifidobacterium* sample. The findings of previous research and that of the current study suggest that in the *Prevotella*-dominant enterotype, consumption of a diet rich in betacyanins may not be effective for SCFA production, as it could potentially hinder the growth of *Bifidobacterium* and lead to a decrease in *Prevotella*. Conversely, *Bifidobacterium*-dominant enterotypes are expected to produce higher SCFAs because *Bifidobacterium* grows more abundantly than other enterotypes. Meanwhile, RP-treated samples showed higher SCFA production than BP-treated samples, which was believed to be the result of the action of various phytochemicals other than betanin contained in RP.

Previous studies have shown that different microbes correlate with SCFAs depending on the enterotype. Fang et al. [63] reported that following in vitro fecal fermentation with *Lactobacillus parabuchneri*, the *Faecalibacterium*-enterotype group was positively correlated with SCFAs with *Faecalibacterium*, *Anaerostipes*, *Coprococcus*, and *Butyricicoccus*, and the *Escherichia/Shigella*-enterotype group was positively correlated with *Streptococcus*, *Bacteroides*, and *Sutterella*. A study on the fecal fermentation of bacteriocins showed that the positive association between gut microbiota and SCFAs was more pronounced in the *Prevotella* enterotype compared to other enterotypes [42]. In this study, SCFA-producing bacteria that were increased by RP and BP were positively correlated with SCFAs; however, bacteria that were increased only in certain subjects were not positively correlated with SCFAs in other subjects. For example, *Ligilactobacillus* and *Veillonella*, which increased in the S1-*Phocaeicola* sample, were positively correlated in the S1-*Phocaeicola* sample, whereas *Coprococcus* and *Alistipes*, which increased in the S2-*Prevotella* sample, were positively correlated in the S2-*Prevotella* sample. In addition, *Anaerobutyricum*, which increased in the S3-*Bifidobacteirum* sample, was positively correlated only in the S3-*Bifidobacteirum* sample. Therefore, these results suggest that the gut microbiota involved in SCFA production, which was altered by RP and BP, differed according to enterotype.

Microbial metabolic activities were predicted using PICRUSt2. At KEGG level 2, various metabolisms are altered after RP and BP fecal fermentation, confirming that “metabolism” is the main microbial metabolic activity. In particular, carbohydrate metabolism was significantly increased in the S3-*Bifidobacterium* group. Starch, sucrose metabolism, and glycolysis/gluconeogenesis were increased in both RP and BP, whereas pyruvate and galactose metabolism were increased only by RP and BP, respectively. Dhananjayan et al. reported that oral administration of betanin increased gluconeogenic enzymes [64]. The glycolysis/gluconeogenesis pathway produces pyruvate and oxaloacetate, the precursors of SCFAs, from alpha-d-glucose-6-phosphate, the end product of starch, sucrose, and galactose metabolism [65]. Although mass spectrometry-based microbial metabolome analysis is needed, these results show that the S3-*Bifidobacterium* sample had useful changes in gut microbial metabolism following RP and BP compared to the other samples. These results indicate that the microbial metabolic activities altered by RP and BP may differ between enterotypes, especially those related to SCFA generation.

This study has some limitations. A key limitation is the small sample size, with only one subject representing each enterotype (*Phocaeicola*, *Prevotella*, and *Bifidobacterium*). This restricts the generalizability of our findings and reduces statistical power. Additionally, there is potential variability in responses among different populations, which could further impact the applicability of our results. Future research should involve larger and more diverse cohorts to better capture these differences. Another important consideration is that our study relied on in vitro fecal fermentation models, which cannot fully replicate the complexities of gut microbiota interactions in vivo. While useful for examining specific responses, these models do not account for factors such as host immune interactions and gastrointestinal dynamics. Furthermore, although it has been reported that gut microbiota can bioconvert red beetroot and betanin, our study did not confirm this bioconversion. Therefore, follow-up in vivo studies and mass-spectrometry-based catabolite and microbial metabolomics analyses are essential to gain a more comprehensive understanding of the effects of RP and BP on gut health according to enterotype.

Nevertheless, these results provide information on the enterotype-specific effects of RP and BP in modulating gut microbiota and SCFA production. To our knowledge, this is a pioneering in vitro study investigating enterotype-specific changes in gut microbiota and SCFA production in response to RP and BP. By linking these alterations to variations in microbial composition, our findings enhance the understanding of personalized nutrition strategies aimed at optimizing gut health through tailored dietary interventions. This research underscores the importance of enterotype differentiation in the development of functional foods targeting gut microbiota and SCFA production. Additionally, as we consider the implications of enterotype-specific responses to RP and BP, the interplay between host health and gut microbiota underscores the need for personalized medicine, as broad interventions often lack specificity. Advancements in nanocarriers could enhance targeted delivery, enabling the co-encapsulation of probiotics and prebiotics for more effective, tailored treatments [66].

## 5. Conclusions

Thirty Korean subject enterotypes were divided into *Phocaeicola*, *Prevotella*, and *Bifidobacterium* enterotypes, and the effects of RP and BP as prebiotics on the gut microbiota differed depending on the enterotype. The S3-*Bifidobacterium* sample showed a significant decrease in the Chao index and a different trend in gut microbiota changes in the NMDS compared to the other samples. The increase in SCFAs was greatest in the S3-*Bifidobacterium* sample, whereas the S2-*Prevotella* sample showed a smaller change in SCFAs. In addition, our results showed that bacterial and microbial metabolic activities related to SCFAs were different for each enterotype. These results indicate that RP and BP have enterotype-specific responses in the gut microbiota and SCFA production.

## Data Availability

The data generated in this study are presented in this paper. Further details are available upon request.

## Supplementary Materials

The following supporting information can be downloaded at: https://www.mdpi.com/article/10.3390/life14111391/s1, Figure S1: Taxonomy composition of 30 subjects at the phylum level. The *, #, and & indicated significantly different abundance in each group (*; *Phocaeicola* type, #; *Prevotella* type, &; *Bifidobacterium* type). Significant different relative abundance analysis was examined using LEfSe (*p* < 0.05, LDA score > 3); Figure S2: Tree analysis for gut microbiota clustering of 30 subjects and the selected subjects; Figure S3: Taxonomy composition of selected subjects at the phylum level. The *, #, and & indicated significantly different abundance in each group (*; S1-*Phocaeicola*, #; S2-*Prevotella*, &; S3-*Bifidobacterium*). Significant different relative abundance analysis was examined using LEfSe (*p* < 0.05, LDA score > 3); Figure S4: Effects of RP and BP on Predicted microbial metabolic activities by enterotype at Level 2 of KEGG pathway (*p* < 0.01). (A) S1-*Phocaeicola*, (B) S2-*Prevotella* and (C) S3-*Bifidobacterium*. BLK, negative control (without fermentable substrate); RP, red beet powder; BP, betanin pigment; Table S1: General characteristics of subjects according to the enterotypes; Table S2: Taxonomy composition at the phylum level after fecal fermentation; Table S3: Relative abundance of *Phocaeicola*, *Prevotella*, and *Bifidobacterium* after fecal fermentation.
