# Supplementary material for: Enterotype-Specific Effects of Red Beetroot (Beta vulgaris L.) Powder and Betanin on Human Gut Microbiota: A Preliminary Study Based on In Vitro Fecal Fermentation Model
Source: Life (Basel). 2024 Oct 29;14(11):1391. doi: 10.3390/life14111391 (PMC11595470; doi:10.3390/life14111391)
Supplement: Supplementary file 1 [file life-14-01391-s001.zip › life-3229178-supplementary.pdf]

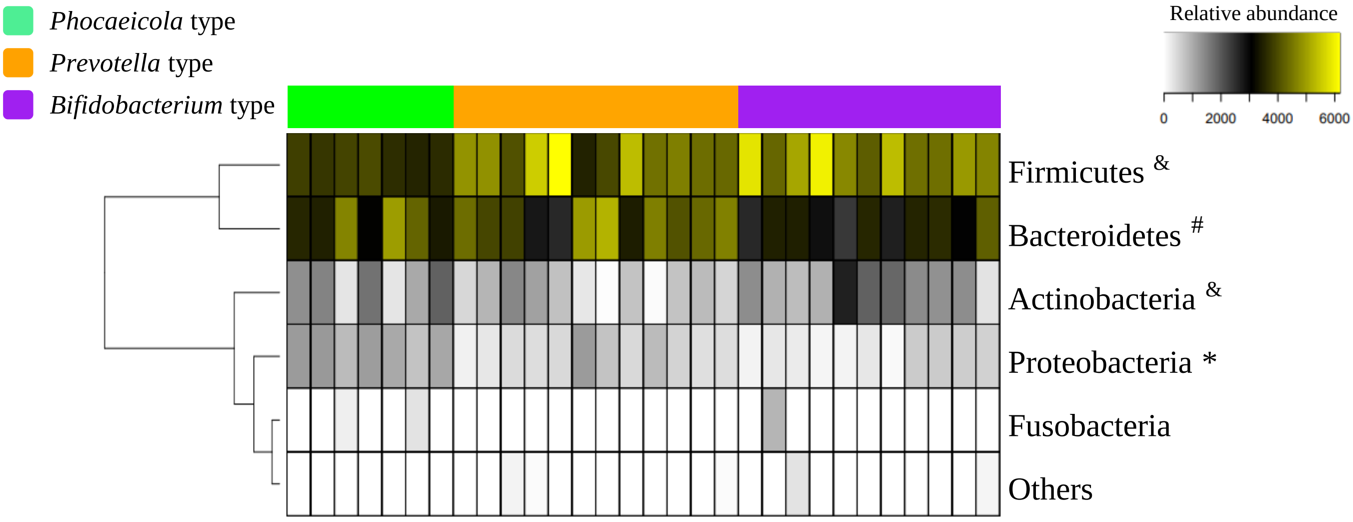

Figure S1. Taxonomy composition of 30 subjects at the phylum level. The *, #, and & indicated significantly different abundance in each group (*; *Phocaeicola* type, #; *Prevotella* type, &; *Bifidobacterium* type). Significant different relative abundance analysis was examined using LEfSe ($p < 0.05$, LDA score > 3).

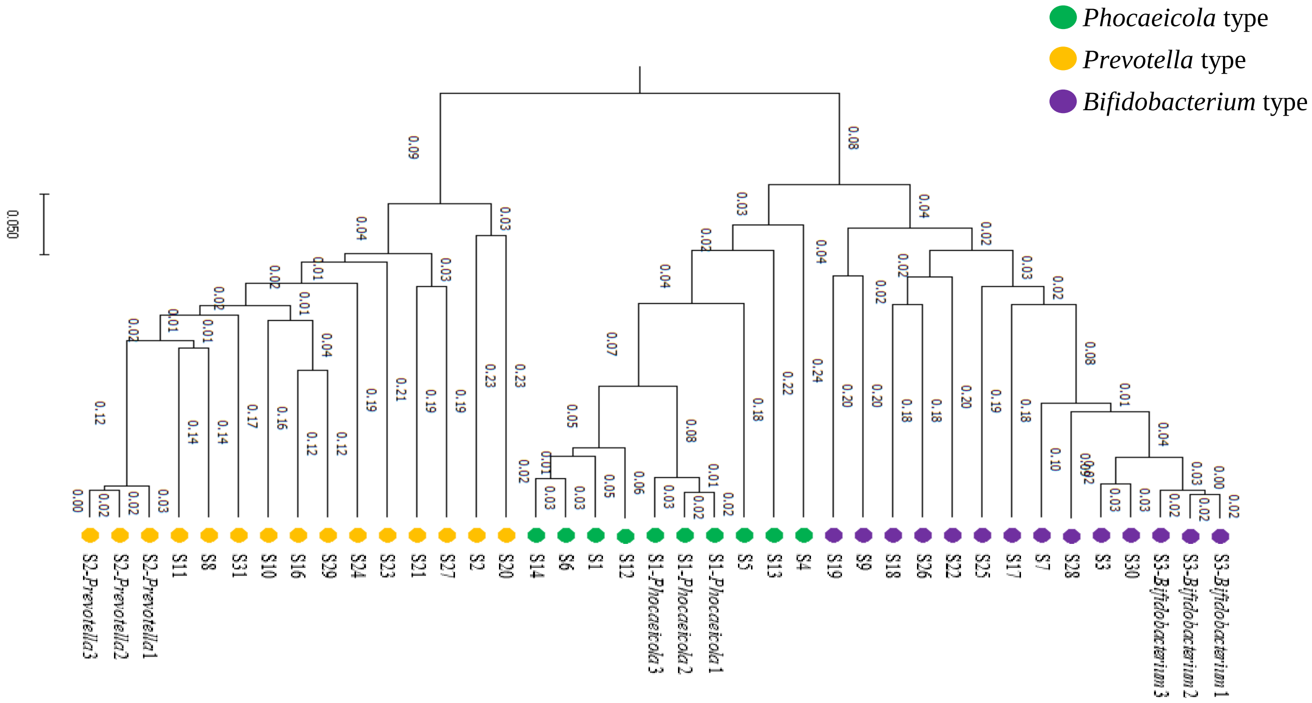

Figure S2. Tree analysis for gut microbiota clustering of 30 subjects and the selected subjects.

Table S1. General characteristics of subjects according to the enterotypes

Characteristics	*Phocaeicola* type (n=11)	*Prevotella* type (n=12)	*Bifidobacterium* type (n=7)
Gender (M:F)	6:5	6:6	4:3
Age (years)	30.5±7.7 [a]	30.1±11.0 [a]	30.8±4.4 [a]
BMI (kg/m2)	23.0±3.52 [a]	23.3±2.7 [a]	24.0±4.0 [a]

BMI: body mass index, a>b; a significant difference at $p < 0.05$, Significant analysis were analyzed using duncan test

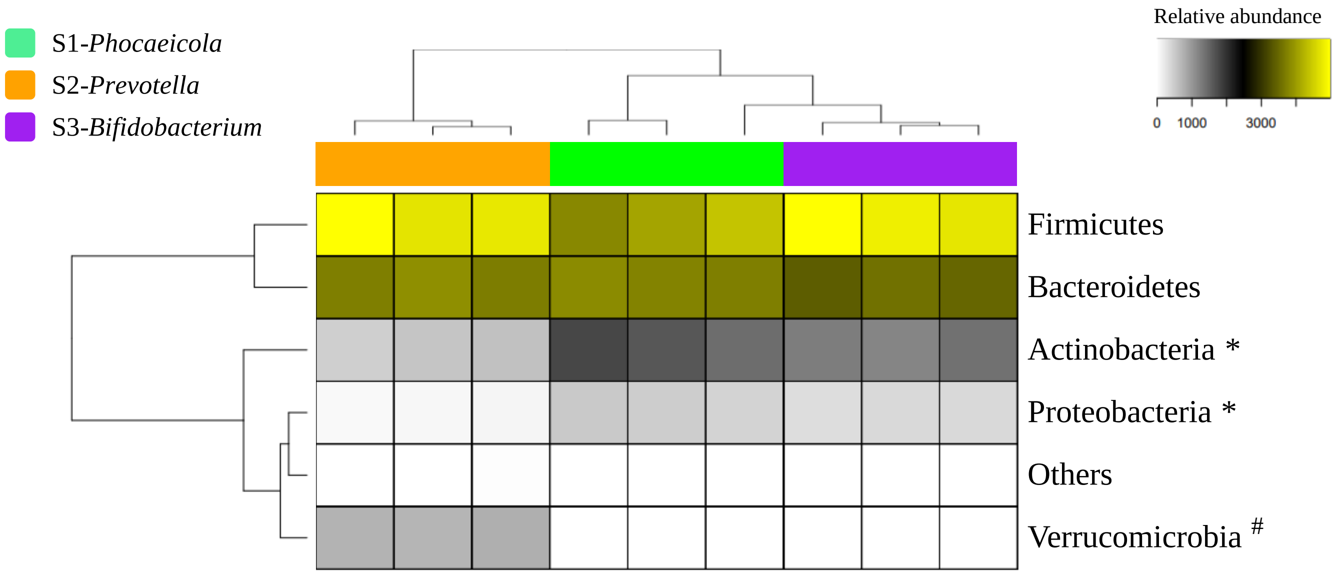

Figure S3. Taxonomy composition of selected subjects at the phylum level. The *, #, and & indicated significantly different abundance in each group (*; S1-*Phocaeicola*, #; S2-*Prevotella*, &; S3-*Bifidobacterium*). Significant different relative abundance analysis was examined using LEfSe ($p < 0.05$, LDA score > 3).

Table S2. Taxonomy composition at the phylum level after fecal fermentation

Taxonomy	S1-*Phocaeicola*			S2-*Preovtella*			S3-*Bifidobacterium*		
	CTL	RP	BP	CTL	RP	BP	CTL	RP	BP
Firmicutes	39.2±1.2	38.3±0.9	36.4±1.8	47.1±4.3	48.8±2.2	47.3±1.5	44.3±0.2	46±0.5	45.2±1.6
Bacteroidetes	36.8±0.8	35.9±0.7	37.1±1	34.2±1.9	30.8±1.8	31.5±1.2	36.8±0.7	35.3±1	34.9±2.3
Actinobacteria	11.7±1.4	13±1 *	14.8±0.7 *	5.6±0.2	9±0.4 *	10.2±0.2 *	9.5±0.6	11±0.7 *	13.3±1.4 *
Proteobacteria	12.2±0.2	12.9±0.3	11.7±0.6	1.1±0.2	1.4±0.1 *	0.6±0.1 *	9.4±0.3	7.8±0.6 *	6.6±0.3 *
Verrucomicrobia	0.0±0.0	0.0±0.0	0.0±0.0	11.9±2.4	9.9±0.7	10.3±0.2	0.0±0.0	0.0±0.0	0.0±0.0
Others	0.0±0.0	0.0±0.0	0.0±0.0	0.1±0.0	0.0±0.0	0.1±0.1	0.0±0.0	0.0±0.0	0.0±0.0

Significant different relative abundance analysis was examined using the liner discriminant analysis effect size (LEfSe).
* indicate significant difference between CTL ($P < 0.05$)

Table S3. Relative abundance of *Phocaeicola*, *Prevotella*, and *Bifidobacterium* after fecal fermentation

Taxonomy	S1-*Phocaeicola*			S2-*Prevotella*			S3-*Bifidobacterium*		
	CTL	RP	BP	CTL	RP	BP	CTL	RP	BP
Phocaeicola	33.1±0.3	28.6±0.6	30.1±0.4	9.4±0.3	9.1±0.6	9.6±0.2	19.7±0.4	15.9±0.5	16.9±0.2
Prevotella	0.0±0.0	0.0±0.0	0.0±0.0	6.8±0.6	4.0±0.3	3.7±0.1	0.0±0.0	0.0±0.0	0.0±0.0
Bifidobacterium	6.0±0.2	8.0±0.1	9.1±0.2	3.0±0.2	4.8±0.2	5.8±0.4	8.9±0.4	11.0±0.5	12.3±0.4

Significant different relative abundance analysis was examined using the liner discriminant analysis effect size (LEfSe).
* indicate significant difference between CTL ($P < 0.05$)

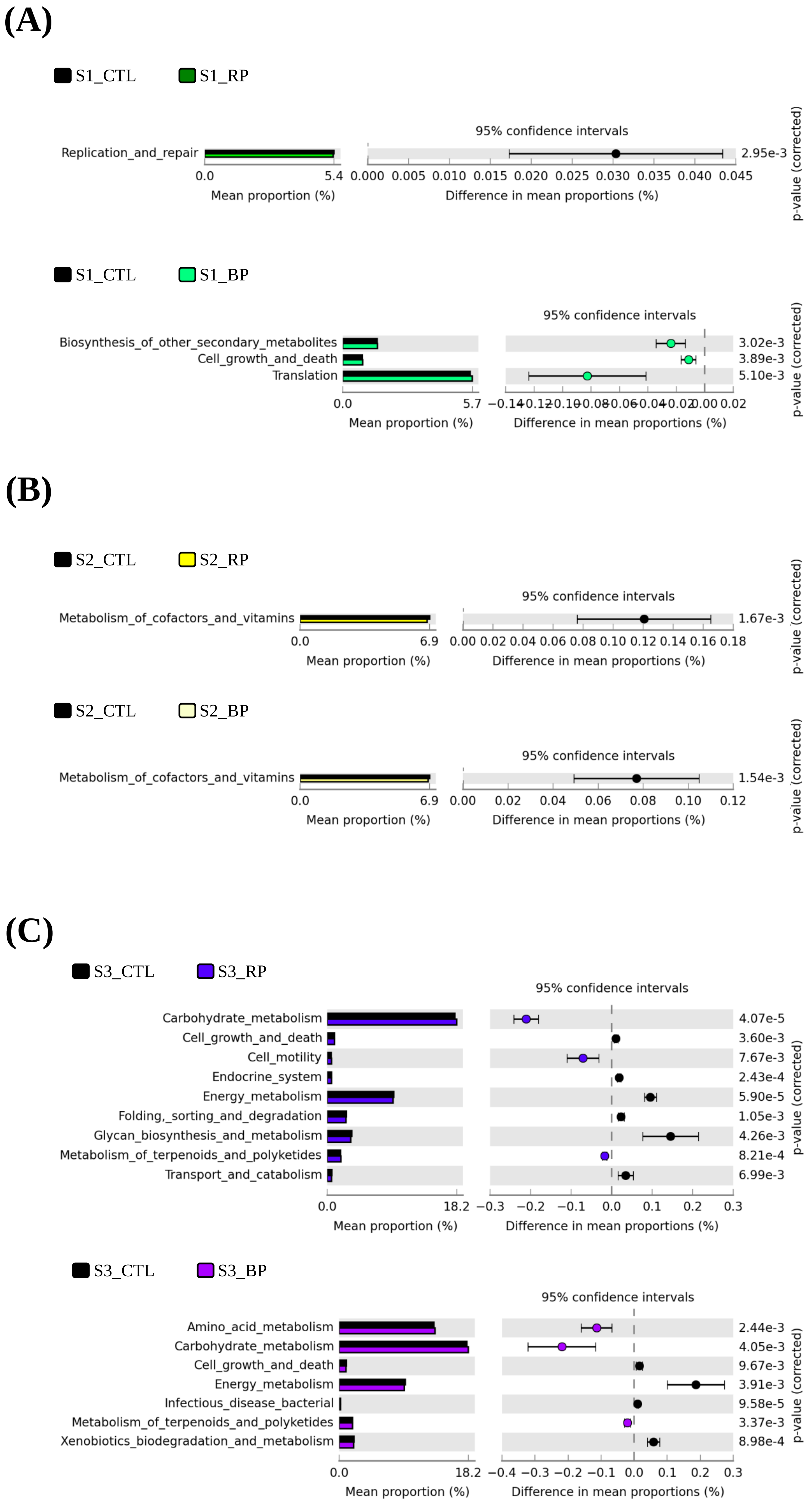

Figure S4. Effects of RP and BP on Predicted microbial metabolic activities by enterotype at Level 2 of KEGG pathway ($p < 0.01$). (A) S1-*Phocaeicola*, (B) S2-*Prevotella* and (C) S3-*Bifidobacterium*. BLK, negative control (without fermentable substrate); RP, red beet powder; BP, betanin pigment.
